# “Be an ambassador for change that you would like to see”: a call to action to all stakeholders for co-creation in healthcare and medical research to improve quality of life of people with a neuromuscular disease

**DOI:** 10.1186/s13023-019-1103-8

**Published:** 2019-06-07

**Authors:** Anna Ambrosini, Ros Quinlivan, Valeria A. Sansone, Ingeborg Meijer, Guus Schrijvers, Aad Tibben, George Padberg, Maarten de Wit, Ellen Sterrenburg, Alexandre Mejat, Alexandra Breukel, Michal Rataj, Hanns Lochmüller, Raffaella Willmann, on behalf of the 235th ENMC workshop study group, Anna Ambrosini, Dimitrios Athanasiou, Nathalie Bere, Fabiola Bertinotti, Alexandra Breukel, Filippo Buccella, Nic Bungay, Caroline Daly, Mencía de Lemus, Maarten De Wit, Ettore Galluccio, Carsten Gamroth, Nathalie Goemans, Gábor Gömöri, Mats Hansson, François Lamy, Erik Landfeldt, Anne Lennox, Hanns Lochmüller, Hank Mansbach, Elena Mazzone, Ingeborg Meijer, Alexandre Méjat, Anneke Mels, Lucia Monaco, George Padberg, Holly Peay, Ros Quinlivan, Jes Rahbek, Marco Rasconi, Michal Rataj, Françoise Salama, Valeria Sansone, Ulrike Schara, Guus Schrijvers, Ellen Sterrenburg, Aad Tibben, Kimberly Trant, Baziel Van Engelen, Arpad Von Moers, Elizabeth Vroom, Raffaella Willmann, Annelies Zittersteijn

**Affiliations:** 1 Fondazione Telethon, Via Poerio 14, 20129 Milan, Italy; 2 MRC Centre for Neuromuscular Diseases, Institute of Neurology, Queen Square, London, UK; 3 NEuroMuscular Omnicentre (NEMO), Neurorehabilitation Unit, University of Milan, ASST Grande Ospedale Metropolitano Niguarda, Fondazione Serena Onlus, Milan, Italy; 4 Centre for Science and Technology Studies (CWTS), University of Leiden, Leiden, The Netherlands; 5 Spierziekten Nederland, Baarn, the Netherlands; 6 Centre for Human and Clinical Genetics, Leiden University Medical Centre, Leiden, The Netherlands; 7 Department of Medical Humanities, Amsterdam University Medical Centre, Amsterdam, The Netherlands; 8 Prinses Beatrix Spierfonds, The Hague, The Netherlands; 9 University of Lyon, University of Lyon1 Claude Bernard Lyon1, Institut NeuroMyoGene, CNRS UMR5310, INSERM U1217, Lyon, France; 10 European Neuromuscular Centre, Baarn, The Netherlands; 11 Polish Neuromuscular Diseases Association (PTCHNM), Warszawa, Poland; 12 Children’s Hospital of Eastern Ontario Research Institute, University of Ottawa, Ottawa, Canada; 13 Division of Neurology, Department of Medicine, The Ottawa Hospital, Ottawa, Canada; 14 Faculty of Medicine, Department of Neuropediatrics and Muscle Disorders, Medical Center – University of Freiburg, Freiburg, Germany; 15 Swiss Foundation for Research on Muscle Diseases, Cortaillod, Switzerland

**Keywords:** Healthcare, Patient engagement, Patient involvement, Neuromuscular diseases, Co-creation

## Abstract

**Background:**

Patient and public involvement for co-creation is increasingly recognized as a valuable strategy to develop healthcare research targeting patients’ real needs. However, its practical implementation is not as advanced and unanimously accepted as it could be, due to cultural differences and complexities of managing healthcare programs and clinical studies, especially in the rare disease field.

**Main body:**

The European Neuromuscular Centre, a European foundation of patient organizations, involved its key stakeholders in a special workshop to investigate the position of the neuromuscular patient community with respect to healthcare and medical research to identify and address gaps and bottlenecks. The workshop took place in Milan (Italy) on January 19–20, 2018, involving 45 participants who were mainly representatives of the patient community, but also included experts from clinical centers, industry and regulatory bodies. In order to provide practical examples and constructive suggestions, specific topics were identified upfront. The first set of issues concerned the quality of life at specific phases of a patient’s life, such as at the time of diagnosis or during pediatric to adult transition, and patient involvement in medical research on activities in daily living including patient reported outcome measures. The second set of issues concerned the involvement of patients in the management of clinical research tools, such as registries and biobanks, and their participation in study design or marketing authorization processes. Introductory presentations were followed by parallel working group sessions, to gain constructive contributions from all participants. The concept of shared decision making was used to ensure, in discussions, a partnership-based identification of the wishes and needs of all stakeholders involved, and the “ladder of participation” tool served as a model to evaluate the actual and the desired level of patients’ involvement in all topics addressed. A general consensus on the outcome of the meeting was collected during the final plenary session.

This paper reports the outcome of the workshop and the specific suggestions derived from the analysis of the first set of topics, related to quality of life. The outcomes of the second set of topics are reported elsewhere and are only briefly summarized herein for the sake of completeness.

**Conclusions:**

The neuromuscular community proved to be very active and engaged at different levels in the healthcare initiatives of interest. The workshop participants critically discussed several topics, providing practical examples where different stakeholders could play a role in making a change and bridging gaps. Overall, they indicated the need for education of all stakeholders for better communication, where everyone should become an ambassador to promote real change. Support should also come from institutions and healthcare bodies both at structural and economic level.

## Background

The European policy concepts of “open science”, “co-creation”, and “responsible research and innovation” define new approaches in the processes of strategic research & development and decision making in which all involved stakeholders, including citizens, contribute to reach conclusions that apply to a broader range of expectations [1]. In practice, it means it is pivotal for science to reach out to stakeholders and engage with them in order to speed up innovation and knowledge to increase the wellbeing of citizens and patients. Co-creation means collaborative generation of knowledge, which in health research relates to the active involvement of patients in experience-based study design of patient-centered health services, research outcomes and clinical investigations [2–4]. The need to engage patients has been solicited at several levels, from setting political and research agendas to building networks to share their experiential knowledge and perspective with academics, clinicians and industry [5–7]. Despite the fact that much has been conceptualized about patient participation (and there is growing evidence of its implementation), much still needs to be done to consolidate practice, explore models of participation and provide clear evidence of its benefits and value [4, 8]. For patients and Patient Organizations (POs) this is a time to discuss if and when they want to participate and how they can contribute to a co-creation process for better-tailored medical interventions.

In the neuromuscular disease (NMD) field, these approaches have long been embraced and supported by the European Neuromuscular Centre (ENMC) [9], a European foundation of empowered NMD POs, whose mission is to promote research and improve quality of life (QoL) of people with NMD. Through the sponsorship of highly focused workshops, the ENMC encourages and facilitates communication and collaboration in the NMD field by promoting interaction among experts (researchers, clinicians, other healthcare professionals, regulators, etc.), including patient representatives [10, 11]. In 2018, the ENMC celebrated its 25th anniversary of activity by engaging its main stakeholders in a special workshop to understand how the NMD patient community impacts upon the healthcare and science co-creation scenario. The workshop took place in Milan (Italy) on January 19–20, 2018 and involved 45 participants from 15 countries, including representatives of POs, clinical researchers, industry and regulatory authorities.

The “Shared Decision Making” (SDM) concept [12–14] and the “participation ladder” model [15, 16] were used as working tools to set the stage for discussion about the position of patients affected by NMDs with respect to their active participation in healthcare and research initiatives.

### Why promote SDM?

SDM has been defined as: “an approach where clinicians and patients share the best available evidence when faced with the task of making decisions, and where patients are supported to consider options, to achieve informed preferences” [13].

In practice SDM implies effective communication between health professionals and their patients about the options of prevention, screening, diagnostic tests and available treatments for their disease, including the choice of not intervening. Through a structured dialogue, medical doctors promote patients’ knowledge of the existing opportunities and a correct perception of benefits versus risks. Moreover, they work towards understanding values and preferences of patients and their families with respect to the available options, taking into consideration barriers and facilitators that influence their practical implementation. Joint decisions derived from better-informed patients lead to reassurance and confidence regarding the choices made and makes patients more confident about their treatment options, ultimately leading to better compliance. Saving money is not a primary objective, but this can nonetheless be one of its consequences. SDM is particularly relevant when uncertainty is high, i.e. when all the available options have drawbacks or when no evidence is available of what the best option might be [13, 14, 17].

### The ladder of participation – real involvement or tokenism?

The concept of a ladder of participation model goes back to 1969, when Sherry Arnstein proposed it to discuss the extent of citizens’ power in determining the end product [15]. She envisaged three broad levels, ranging from non-participation, tokenism, to real power and full control, with different degrees, or rungs, for each of these categories. This concept has been used to investigate the level of engagement and type of role of patients and citizens play in healthcare decision making [18, 19]. Moving beyond the classical, normative structure suggested by Arnstein, Tritter and McCallum [16] argued that when health issues are concerned, the ultimate goal of patients is not necessarily to reach full power and control, as it is “the process rather than the outcome that has the greatest potential for changing organizational culture”. In other words, they suggested that all the different levels of participation in the higher part of the ladder are relevant. In fact, gaining full power of the governance aspect is not always the target, since decision making in health research may derive from the harmonization of different viewpoints from different stakeholders. The role of patients’ may vary depending on the context, their competences and the resources available. Studies from Abma and collaborators have shown the variety of roles and conditions under which patients or their representatives have been able to effectively contribute to research practices [20]. It must be born in mind that partnering with patients and families for collaborative projects should be seen as a means to improve healthcare, and participation is not the ultimate goal per se [4].

Aligned with these views, in this workshop we wanted to investigate the experience of the NMD community regarding patient – clinician partnerships for both healthcare delivery and research. In particular, we wanted to understand where and to what extent patients can play an active role in these processes, and also to understand what they hope to gain through their involvement. Therefore, we adopted the model as in de Wit [19], which considers different levels of involvement.

**Fig. 1:**
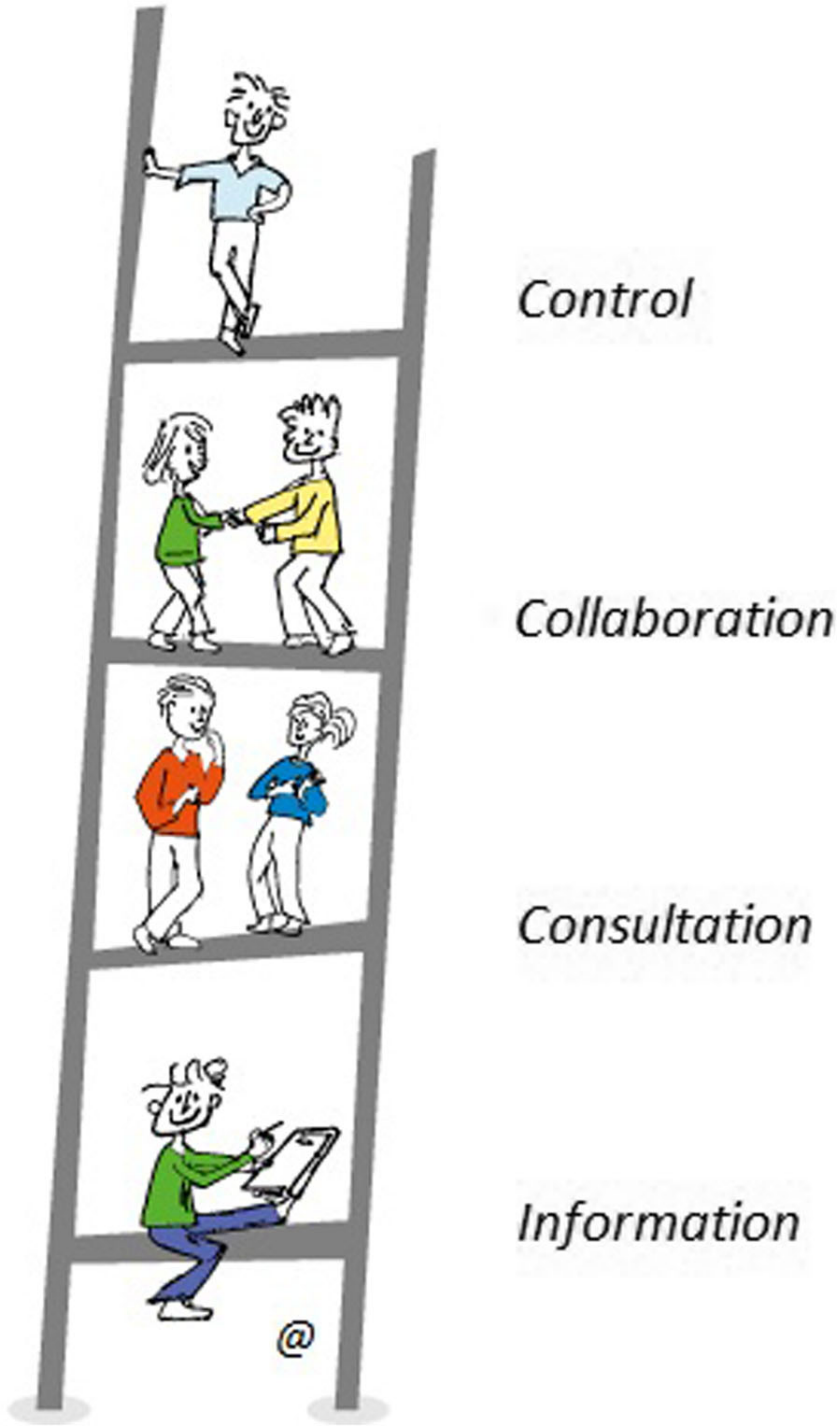
Levels of patient involvement along the participation ladder (A. Ambrosini; modified from [19])

The lowest, non-participation level is limited to when the information given to the patient by the professional is about decisions that have already been made. The level of symbolic consultation is reached when the patient’s advisory role is requested only after or at the end of the decision process, thereby limiting the possible influence of the patient on the process. At ‘the collaboration level’, patients provide information on their condition and medical needs and give advice to clinicians. This role as partners in the process of production of knowledge and co-creation of new health research models and tools involves, for instance, providing input about the development of new functional outcome measures, or into studies targeting activities of daily living, or on the design of technological devices. When the ‘control’ level is reached, patients take the initiative and hold varying degrees of power depending upon their relative contribution and ownership of the process (Fig. 1).

### The ENMC workshop

The SDM model and the participation ladder were used as tools to operationalize participation of the different stakeholders attending the meeting. The main objectives of the workshop were: i) to analyze the current state of play of patient involvement in the NMD field with regard to the topics selected; ii) to identify critical issues and types of intervention; and iii) to identify priority issues from all stakeholders that need to be addressed in practice and how this should be achieved.

The discussion focused upon 6 specific topics related to patient participation in healthcare delivery: topic 1; psychosocial support for families undergoing medical diagnosis and/or prenatal and predictive genetic testing; topic 2; transition from childhood to adulthood; and topic 3; the impact of patient participation in all aspects of medical research (such as QoL, patient-reported outcome measures (PROMs), other outcomes and interventions); topic 4; registries and biobanks; topic 5; design and implementation of clinical trials; and topic 6; interaction with regulatory authorities. Successful examples including individual patient reports were illustrated during plenary sessions, followed by structured group feedback, which engaged all participants to assess critical issues, share their experience, and provide practical inputs.

The content of this paper reflects the outcome of workshop topics 1–3 and, in particular, it focuses on the QoL-related healthcare session (topics 1 to 3). This includes the specific needs identified and priority areas for implementation including: where and how patient involvement could make a difference and how this might best be achieved. The outcomes of topics 4–6 are reported elsewhere and for this reason will only be briefly mentioned [21].

## Methods

### Contents and structure of the workshop

The content of this workshop was set up by the Executive Committee of the ENMC together with its Research Director. The identification of the two working models, SDM and participation ladder, was based on extensive preliminary review of the literature regarding patient engagement. The choice of the six topics that were investigated derived from ENMC members’ knowledge of the field, strategic analysis of the outcomes of ENMC sponsored workshops, evaluation of the broad international NMD network activities, and consultation with the ENMC Research Committee members.

**Fig. 2:**
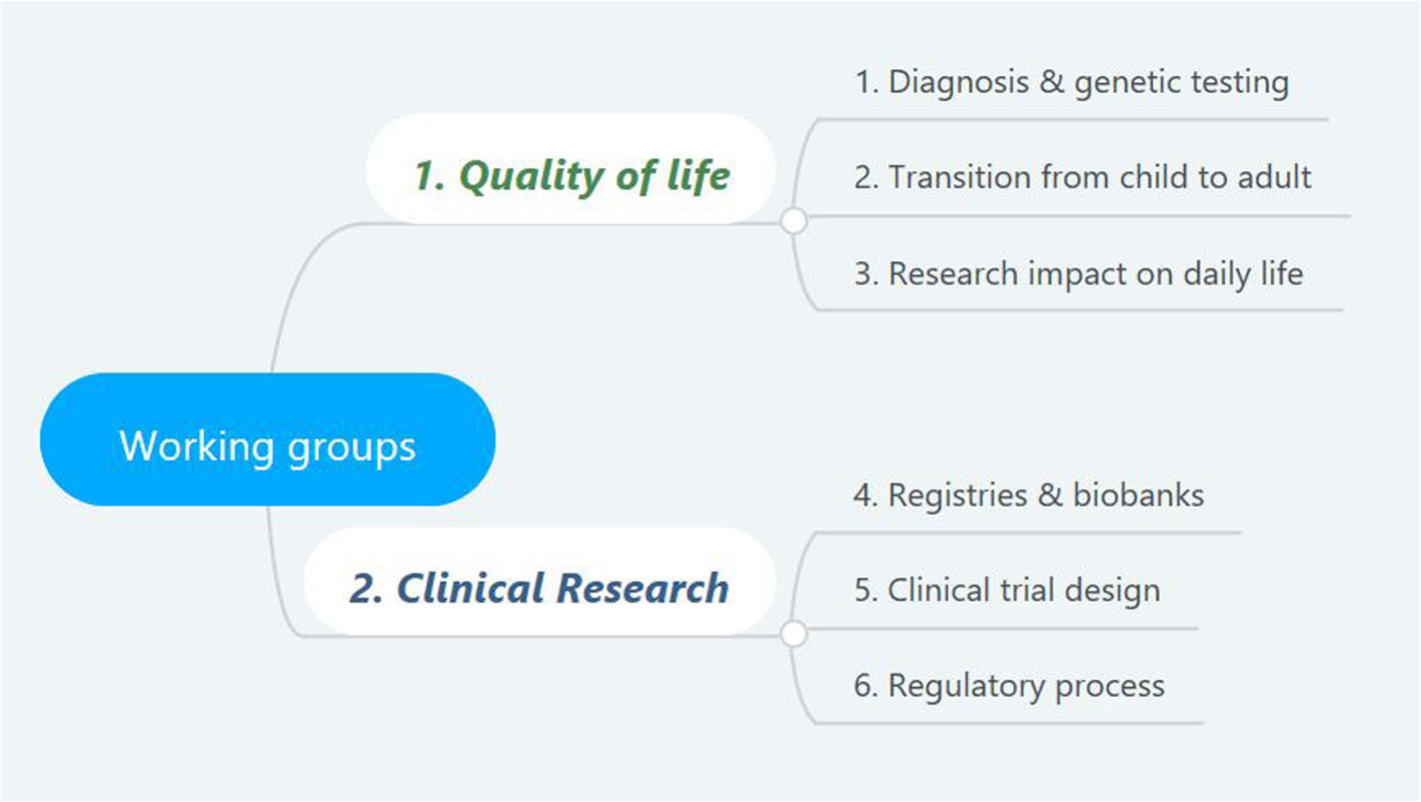
Structure of the workshop’s working groups

The workshop structure consisted of plenary lectures, aimed at setting the groundwork for the discussion, followed by parallel working groups that involved all participants in the discussion on the specific topics (Fig. 2).

In order to provide the audience with the instruments to be used during the discussion, Guus Schrijvers introduced the SDM concept and Ingeborg Meijer illustrated the ladder of participation model. She also provided the participants with operational instructions about the workshop.

Session 1, “SDM in Quality of Life and psychosocial support”, consisted of three plenary presentations focused on topics 1 (genetic screening and diagnosis), 2 (transition from childhood to adulthood), and 3 (impact of medical research in QoL, aimed at providing: evidence-based examples of effectiveness of patient involvement in healthcare, practical elements and ‘real life’ experience of the benefits of being engaged.

Session 2, “SDM in clinical research and trial design” was introduced by the ENMC Research Director, George Padberg, who adopted the principles of SDM and patient involvement in research to investigate their possible roles in the critical scenario represented by the implementation of personalized medicine in the NMD field, and its consequences for the future. Three plenary presentations followed, targeting topics 4 (registries and biobanks), 5 (clinical trials), and 6 (regulatory and consenting processes).

After each session of talks, all participants were divided into three parallel discussion groups, each addressing one of the topics.

During Session 3, the outcomes of the six working groups were presented in a plenary session and discussed with the whole workshop, and a consensus on the key messages was reached among all participants.

In order to make the results of the workshop easier to follow, a brief highlight of each specific topic of Session 1 is reported, followed by the outcome of the respective working group.

### Terminology

The terminology used in this paper refers to the National Institute for Health Research (UK) definitions [22] namely:

- patient and public involvement is the development of partnerships between patients, carers or other members of public and researchers. Such partnerships are made in order to influence what research is done, how, and what happens to the results;
- patient and public engagement is the sharing of information and knowledge about research by professionals, such as during open days, science festivals or through newspapers or other media;
- patient and public participation is the recruitment of patients or others to take part in clinical trials or other research studies.

This terminology is adopted irrespective of the participation ladder connotations and is meant to unambiguously define the type of contribution by patients in their partnership with professionals for medical research.

## Results

### Session 1 - SDM in quality of life in NMDs: psychosocial support

Health-related QoL is a broad concept that defines the perceived quality of an individual’s daily life from different perspectives, which refer to physical, emotional and social aspects, according to the original World Health Organization definition [23]. Specific instruments have been developed to assess patients’ well-being or lack thereof, such as PROMs and QoL surveys. In general, these tools are meant to capture directly from patients the impact on their daily life of the disease and, where applicable, of a treatment or a technological device. The definition and implementation of these instruments is based on the fruitful collaboration among various stakeholders (patients, clinicians, biomedical engineers, etc.). At the workshop, participants discussed how to promote better QoL in people living with a NMD condition, examining ethical and psychological issues related to significant phases in their lives, from the diagnostic process to the transition from childhood to adolescence and adulthood, how to contribute to the development of PROMs and clinical studies targeting daily life issues. Emphasis was put on the contribution of patients in defining research priorities, as well as any related challenges and how they might be overcome.

#### Topic 1: SDM in helping patients and families go through genetic testing (prenatal and predictive) and/or diagnosis

##### Introductory talk 1 – knowledge is power

Topic 1 was introduced by Aad Tibben. He presented a summary of the positive experience of the European Huntington’s disease (HD) network (EHDN) [24] to highlight the importance of the synergy among people affected by a rare disease and their relatives, clinicians and researchers and the need for them to work together to advance research and improve quality of care. In particular, he addressed the psychological needs related to diagnosis and/or predictive testing for HD, two clinical services with different purposes that bring very different ethical issues. Aad Tibben demonstrated how the excellent interaction that occurs within the EHDN allowed them to develop important recommendations for genetic counselling within families [25, 26]. While presenting data on this experience, Tibben discussed several aspects related to the psychological burden of affected people and their relatives that may also apply to other serious rare genetic diseases, including many NMDs, and indicated how good practice of SDM is important to give patients and their families the skills to deal with these difficult issues.

He cited the philosophical motto “Knowledge is power”, adapting it to the current field of genetics, which has seen tremendous technological advances in recent years. Easy access to the world-wide web and social media has enabled people to gain detailed information about their specific condition when available. Thus, mutual dependency of all stakeholders and SDM are essential to make this information powerful and fruitful. Diagnostic or predictive testing together with good SDM allow for the promotion of patient autonomy, individually tailored decision-making, environmental mastery, and ultimately improvement of QoL. Tibben pointed out that this is the future of medicine and it requires adaptation of all stakeholders involved, to better define concepts on SDM, improvement of stakeholders’ discourse, definition of responsibilities and boundaries, formalization of arrangements and structures, and legal and ethical considerations.

##### Panel discussion

This working group acknowledged that, indeed, several of the issues and hurdles described by Aad Tibben are also common experience within the NMD community, including genetic diagnosis of an affected person and predictive testing in siblings or other relatives. Many NMDs are congenital in onset and affect primarily children and the group discussed if or when it would be the best time to test a younger sibling who might be either unaffected or pre-symptomatic when no therapeutic options are available. This scenario is starting to change for Duchenne muscular dystrophy (DMD) and spinal muscular atrophy (SMA) where therapeutic options are now available [27–29]. These developments call for a change in medical practice to identify pre-symptomatic children and, in particular, to initiate programs for neonatal screening. Indeed new care guidelines developed by specialists with the PO support have been released [30–33]. The POs emphasized the importance of families knowing their specific DNA mutation and for them to receive guidance on the best medical options for their QoL through a well-structured SDM process.

From these observations, the panel concluded there is a strong need to create awareness and effective communication between doctors and patients/parents, and to identify specific roles and competencies on both sides (Table 1A).

**Table 1:** Identification of challenges to address and gaps to bridge for a more effective involvement of people living with a rare disease condition in healthcare and medical research (PO: patient organization, SDM: Shared decision making, EUPATI: European Patients’ Academy on Therapeutic Innovation, NMD: neuromuscular disease)

	Educational changes	Cultural changes	Structural changes
A. Genetic testing and screening/diagnosis	• Empower patients and train them on the challenges of genetic testing or screening programs • Identify leaders who can take the role on specific initiatives • Teach patients and parents to ask the right questions • Avoid making decisions at an individual level, but rather opt for groups to get together at either national or European levels to move things forward • Set up support groups • Launch surveys in the patient population.	• Map the best practices from the different countries and share results • Explore the experience of POs in other rare disease fields • Have a proactive, forward looking approach to upcoming therapeutic options, and take actions according to the opportunities ahead • Stimulate peer-to-peer discussion, create awareness together with the existing POs • Inform patients/families about the existence of POs of reference.	• Create more dialogue with POs, involve patients or PO representatives before implementing screening (why, when, definition of the type of support) • Set up a diagnosis/screening group involving the experts and the disease-specific POs • Train doctors on SDM regarding how best to create dialogue and involve patients • Proactively stimulate workshops about this topic.
B. Transition childhood-adulthood	• Educate physicians, healthcare practitioners, young people, parents, care-givers on SDM • Provide unbiased, validated information on diagnosis, treatment options, sexuality, family planning and life choices • Involve young people and POs as experts to understand educational needs and develop fitting materials (co-creation) • Use appropriate language: right level of difficulty and “people living with....” instead of “patients with...” • Coach all persons involved and create ambassadors among patients, parents and caregivers to show the value of behavioural changes. • Facilitate support by peers and the patient communities to exchange experiences and information.	• Focus on life goals instead of disease management only • Progressively involve children in decision making and facilitate their conversation with physicians (even without parents) • Address patients as a person not as their disease’s needs only • Promote increase in social participation • Enable patients to live independently (in the family, at school, at work) and become autonomous adults • Increase awareness of employability of young people with NMD • Raise confidence and self-esteem of young patients, involve them in team sports and encourage a network of friends.	• Establish specialized centres for paediatric to adult care with multi-disciplinary teams to develop expertise and understand more of the natural history, also learning from other rare diseases • Include young people in the design of services • Use influence and implementation power of organisations like EURORDIS, European Patient Forum, EUPATI • Develop (international) standards of care guidelines for transition and adult care, including the use of SDM • Implement transition programmes • Establish financial support for transition programmes and patient advocate groups • Health insurances, funders and governments to cover costs for all-inclusive (regardless of physical disability) social participation programmes (e.g. summer camps) and decision aids and tools. • Stimulate a dedicated workshop to this topic.
C. Research with impact on quality of life	• Inform about centers of expertise where patients may receive adequate information • Consider challenges in SDM: - Personal barriers (age, communication skills, information gaps on technical terms) - Misinformation in the lay communication and social media - Indirect SDM mediated by the caregiver’s vision and interaction.	• Patients’ interests can create a change the way society works • The introduction of patient panels in commissions and advisory boards may go through pilot rounds to provide evidence of their usefulness • Patient groups’ contribution to clinical study design may positively impact on regulatory agencies’ evaluation of clinical studies.	• Encourage patient-close research, explore patients preferred measurements as new tools • Reserve more time to set research plans with involvement of patients opinion; physician-patient interaction should be based more on the person than on the disease • Train next physician generation in SDM as common practice.

As a starting point, the group also identified specific action points including:

- Setting up a diagnosis/screening group involving experts and the disease-specific POs to address opportunities, hurdles and ethical issues related to the different options and time points of genetic testing and prenatal/neonatal screening programs.
- Develop a proactive approach to upcoming therapeutic options, and take actions according to the opportunities ahead.
- Promote awareness about the requisites for predictive testing and launch surveys in the patient population to investigate best practices, identify “do’s and don’ts” and specific wishes of disease groups.
- Proactively seek applications to the ENMC for workshops around these suggestions.

The panel argued that mutual support between patients/families and clinicians is essential to succeed in improving genetic testing procedures and counselling. In particular, POs may contribute to this process by creating awareness and establishing support groups, while doctors can support a peer-to-peer mentoring (i.e. to facilitate discussion on the impact of decisions that may differentially affect QoL). POs recognized their responsibility in promoting change and were persuaded that if patients change, doctors will follow.

#### Topic 2: SDM in transition from child to adolescent, to adult patient

##### Introductory talk – growing in SDM

Ros Quinlivan discussed how to prepare young people for SDM, through a process that needs change from a paternalistic approach for young children to a shared approach for adolescents and finally to informed SDM for older teenagers. She reported the experience of the MDStarNet with boys and young men with DMD [34]. The new population of young adults with DMD is healthier than before and thus there are new issues and expectations arising. Young adults with DMD are more independent and do not want to be considered different from their peers. However, they may not always necessarily understand their condition and the importance of health screening and in some cases may be less aware that they have a life-limiting condition. This can result in a high rate of non-attendance for routine follow-up appointments, sleep studies and cardiac examinations at the care centers. In addition, young patients may be unaware that they can make choices regarding future healthcare planning [35].

Teenage healthcare behaviours impact on outcomes and, in general, a greater rate of risky behaviours are reported in young people with chronic conditions [36–39]. Quinlivan examined the types of support young people need to make decisions. In her experience, these include: i) information: young people can make informed choices only by understanding their condition and the options available to them; ii) autonomy, which needs to be guaranteed to involve them in SDM; iii) coaching and mentorship; iv) support - teenagers need support and guidance from parents, professionals and peers, otherwise they will be anxious and afraid to be involved in SDM.

She illustrated how, with support from the PO Muscular Dystrophy UK and a clinical psychologist, patients attending her service at The National hospital for Neurology and Neurosurgery were given structured peer support to grow their self-confidence in taking over their own decision making. This included: the development of a hospital-wide young persons’ (YP) steering committee to improve services, with representation from young people on the committee, a YP peer support group facilitated by a clinical psychologist; information specific for YP, and an information day on practical issues such as applying for university; getting a driving licence and living independently and working with a disability [40]. Transition should start early, from about 12 years of age with a key worker and clear goal setting together with aspirations for the future. They need knowledge so that they can learn how to manage their own medical needs, such as being able to recall their medication and doses or understanding what each medication is for. In essence, to prepare young people with NMD for SDM, they need to understand their condition, they should be progressively involved in decision making initially with their parents and, as they become more confident, they should be involved in SDM with or without support from their family. POs can play a major role in facilitating this process, for instance by: i) creating support groups together with professional experts, as in the UK example reported above; ii) promoting the development of young patient advisory groups, and iii) structuring focused initiatives with the families as part of their main organization’s activities.

##### Panel discussion

This working group focused the discussion on the transition from childhood to adolescence of young people with DMD, as a paradigm for the topic.

The group acknowledged that enabling children with a chronic condition to acquire proper skills in decision making is indeed relevant for them to better handle their life and become empowered adults. However, this process requires actions at several levels, which involve physicians, healthcare practitioners, parents, and caregivers. Four areas of priority where formulated, namely: 1. Coaching behavioral change; 2. Increase knowledge to enable SDM; 3. Increase role of society to enable participation; 4. Promote changes in healthcare services to meet the complex health needs of this population of YP (Table 1B).

In particular, coaching and support of patients and their parents and caregivers should be promoted, in order to grow self-esteem and confidence in young people and to help them understand the value of behavioral changes and adopt healthy and constructive attitudes into adulthood. SDM should facilitate the discussion on diagnosis, treatment options, sexuality, family planning and life choices, with psychological support offered at every stage.

Relevant material should be produced to address educational needs, involving POs and patients themselves in this co-creation process that generates ambassadors for spreading a cultural change. Financial support for transition programmes and patient advocacy groups should be made available by national or international institutions.

It was also suggested that a specific workshop could help to set the stage for these changes and delve deeper into the conceptual changes required to enable a child with an NMD to acquire the skills for SDM.

#### Topic 3: SDM in research that has major impact on daily life

##### Introductory talk – health care research requires co-creation

Based on his personal experience as a patient representative in the international research community “Outcome Measures in Rheumatology” (OMERACT), Maarten de Wit focused on the role of patient research partners in different phases of health research. A patient research partner is defined as a person with a relevant condition who provides a patient perspective in the research team as an equal collaborator at all stages of the project. As a first example, effective methods for involving patient research partners in research agenda setting were presented. In the UK the James Lind Alliance has supported many POs to prioritize research topics from the perspective of patients [41]. In The Netherlands the Dialogue model was developed as a step-wise approach to elicit research priorities of different stakeholders, and achieve consensus through heterogeneous focus groups that ensure that the voice of patients is heard [42].

A second example followed the strategy of engaging patients as research partners in the development of a PROM for Psoriatic Arthritis [19]. This case study demonstrated that listening to patients is critical to capture all that is important from the perspective of the target population to include in a new QoL questionnaire. It also showed that having patient research partners on the research team guaranteed that the voice of patients was not lost during the final stages of validation. Finally, having different forms of patient and public involvent (PPI) at different stages of the development process, increased the content validity of the final instrument.

Crucial factors of success for the involvement of patient research partners are to: a) involve patient representatives as collaborative partners from the outset; b) ensure the commitment of the research centres’ leaders; c) prepare early career researchers to interact in a constructive way with patients through creating the environment for an SDM culture and by facilitating collaborative partnerships [43].

##### Panel discussion

This group considered that, for effective involvement in developing research relevant to their daily life, patients need to have a good understanding of the real therapeutic opportunities and of how their input could contribute to healthcare improvement. Physicians need to understand the added value of spending more time in SDM, which will be more likely to improve treatment compliance and lead to constructive contributions to research. It was argued that, in general, patients are not fully involved in discussions relating to their condition and the benefits and risks of a certain treatment. Therefore, they are not ‘trained’ to contribute to building knowledge on what is relevant to them. Moreover, care options relevant for a patient may not be taken into account because they are not part of the standardized methods to measure efficacy and, as such, maybe unfamiliar to the physician. Examples were given of the challenge of managing patients who are unable to speak, where care options are more tailored to the caregiver than to the patient’s real needs. These considerations highlighted the key role of SDM and led to an examination of what the challenges are for a positive and fruitful interaction between patients and physicians (Table 1C). It was acknowledged that fruitful communication has to be based on the understanding of the information gaps and on positive interpersonal contacts, centering the discussion more “with the patient” than “against the disease” [44]. All agreed that a cultural shift is needed for all stakeholders. Actions that may be perceived as exploratory in the beginning could become, if successful, accepted and even necessary once they demonstrate their key value for the main objective. It was noted that a methodological evaluation of a successful SDM could be useful to show benefits and encourage its implementation in areas where it is not yet applied.

Finally, the group concentrated on how to address those limitations that impair development of research that really targets patients’ daily needs. Special calls or programs for clinical studies on QoL, with patient involvement on study design and development of proper PROMs, and dedicated funding for patients involvement in research approaches can better address this need and also pave the way for broad applicability [45–47].

### Session B - patient involvement in clinical research, trial design and regulatory affairs

A rapid increase in innovation and emergence of new healthcare technologies has the potential to reshape patient care and disease; i.e. gene therapy aims to repair the direct cause of genetic disease, by introducing genetic material into cells to compensate for mutated or faulty genes. Treating a range of diseases that until now have been incurable may become possible in the near future. In this developing scenario, patients not only want to be informed, but also request to be active players to influence the developments according to their main needs, by promoting services and contributing to research as ‘expert patients’.

This session investigated opportunities and challenges of direct involvement of patients in medical research [21]. On the one hand it was considered how SDM might be applied in research by facilitating patients’ understanding of the implications of their participation for instance in a trial, thus favoring compliance. On the other hand, this session delved more in the participation ladder, by exploring where, in research, the active role and engagement of patients can make the difference. Several examples were described by participants that have been involved in research-related activities at different levels, being members of Eurordis [48], of the European Patients’ Academy on Therapeutic Innovation (Eupati) [49] or the European Patient Forum [50]. They reported their experience of participation at different research steps, from early stages of translational research, up to the actual delivery of a therapy in the ‘real world’, as well illustrated in the literature [7, 51, 52]. There was common agreement that patients and POs can greatly contribute to implement services to research such as registries and biobanks, by not only donating their data or samples, but also playing a role in the governance with high level of power and control. Regarding the design and implementation of clinical trials, patients should be listened to and involved from the very beginning, to provide input regarding the definition of trial outcomes and the design of informed consents tailored on their real need of information, and to contribute to disseminate the information regarding the trial and its results within their community [53, 54]. The relevance of including disease-specific patients in regulatory agencies’ or ethics’ boards was also recognized, with several examples shared by participants that are already engaged in these top level initiatives. Although it was acknowledged that patient representatives are already taking up roles in decision boards, it was pointed out that in some cases their inclusion reflects more a tokenistic approach, without having the proper patient expertise at the decision table [21].

## Conclusions

A growing body of literature emphasizes the relevance of PPI as a means to promote clinical research of great impact for patients’ real needs. The necessity to involve patients in a co-creation process for collaborative generation of knowledge is also urged by international research consortia [55], industry [7, 56] and regulatory authorities (EMA) [51]. However, translating the overall concept into practice still presents several challenges, experienced both by clinical researches and PPI contributors [57]. With this workshop, the ENMC wanted to discuss with its key stakeholders where the neuromuscular community stands in terms of PPI and co-creation in research, where the major gaps are and what approaches could be taken to address them.

Participants witnessed, through several positive examples, that a strong relationship and constructive interaction has been established among all stakeholders in recent years, especially for those disease groups where therapeutic options are now becoming available. Several PO representatives participate in international patient advisory groups, such as Eurordis, Eupati, or the European Patient Forum, indicating awareness of the value of the contribution they can bring to the community.

The workshop also delved into the hurdles and bottlenecks that still exist, addressing QoL of patients and families in specific phases of their life (i.e. at diagnosis or at transition from child to adulthood), or how consolidated is PPI in NMD clinical research. Table 1 of this report summarizes what aspects the participants identified as priorities regarding the topics discussed, and provides practical examples of how POs/patient representatives and professionals could address them. It has to be noted that, on the one hand this list is not meant to be exhaustive of all the hurdles that people living with a NMD condition may experience, while, on the other hand the proposed examples can be generalized and represent a useful approach to be taken also for other rare diseases.

Overall, the group identified three main levels for proactivity (Table 2).

**Table 2:** Key messages of the 235th ENMC workshop

Main levels for proactivity	
• All stakeholders involved in shared decision making require education: - People affected by a neuromuscular disorder- at any age - Families, healthcare professionals and physicians, parents, caregivers, regulators, industry	
• A cultural change is needed and everyone has to act as ambassador for: - Respectful communication among all partners - Independent contribution of all partners - Societal acceptance and support - Pro-active role of all stakeholders, including patients, caregivers and parents	
• Structural, process and legislation changes should be promoted by: - Learning from models developed by other disease societies and patient organizations - Including disease-specific patient representatives in advisory boards and ethics committees - Including patient organizations and patient representatives in every stage of clinical trials - Setting dedicated funding by national and or international institutions to support patient involvement in medical research activities	

First of all, fostering good communication is fundamental to understand one another and set the groundwork for fruitful interactions (the SDM and participation ladder concepts were used as working models). Proactivity, training, and good understanding of the required level of PPI are other key elements and everyone who embraces this view should act as ambassador to make real change.

With this workshop, the ENMC offered the platform to discuss the level of PPI for patients with NMDs and with this report it creates a tool for all stakeholders to help implement the cultural, educational and structural changes at the local level and expand the group of engaged patients. PO representatives were invited at the workshop to express their individual opinion and not to act officially on behalf of their organization; however, their commitment to create awareness by dissemination and outreach of the workshop deliverables at their local level is expected. Clinicians and other professionals attending the workshop also agreed on the conclusions. They suggested to promote these changes in their own environment and implement them in their activities related to research, for instance by including patients in Executive or Safety boards for a trial, and education, e.g. by having patient representatives take part in the dissemination of standards of care.

Finally, it was hoped that such cultural change is also supported by structural and legal requirements, in order to make PPI included in the plans and realistically developed. Although based on the experience of the strong neuromuscular community, these conclusions do not apply only to this specific field; they fit rather well with the considerations recently reported by other experts [58].

## Data Availability

Not applicable. The paper reports the outcomes of a workshop.

## Abbreviations

DMD: Duchenne muscular dystrophy
EHDN: European Huntington’s disease network
ENMC: European neuromuscular centre
EUPATI: European Patients’ Academy on Therapeutic Innovation
HD: Huntington’s disease
NMD: Neuromuscular disease
OMERACT: Outcome measures in rheumatology
PO: Patient organization
PROM: Patient-reported outcome measure
QoL: Quality of life
SDM: Shared Decision Making
SMA: Spinal muscular atrophy
YP: Young Person
